# Technical aspects of laser treatment for acute retinopathy of prematurity under topical anesthesia

**DOI:** 10.4103/0301-4738.71689

**Published:** 2010

**Authors:** Subhadra Jalali, Rajvardhan Azad, Hemant Singh Trehan, Mangat Ram Dogra, Lingam Gopal, Venkatapathy Narendran

**Affiliations:** 1 Smt. Kanuri Santhamma Centre for Vitreo-Retinal Diseases, L V Prasad Eye Institute, Hyderabad, India; 2 Dr. RP Centre for Ophthalmic Sciences, AIIMS, New Delhi, India; 3 Army Medical Corps India; 4 Department of Ophthalmology, PGIMER, Chandigarh, India; 5 Sankara Nethralaya, Chennai, India; 6 Aravind Eye Hospital, Coimbatore, India

**Keywords:** Childhood blindness, cryotherapy, laser photocoagulation, retinopathy of prematurity, topical anesthesia

## Abstract

Retinopathy of prematurity (ROP) is a significant cause of childhood blindness. The criteria for laser therapy have been revised from threshold ROP to include the earlier stage of high-risk prethreshold ROP. Laser photocoagulation is an established technique for the treatment of ROP. However, the detailed procedure and techniques for laser photocoagulation have not yet been published. Adequate and appropriate laser photocoagulation for ROP is different from the application of lasers in adult retinal vascular diseases, and many ophthalmologists need to be trained in this technique if the outreach of ROP treatment programs is to improve. Laser under topical anesthesia has been practiced in India as a preferred modality especially due to logistics and risks of general anesthesia in these pre-term babies. We discuss the details of the technique as practiced at tertiary care ophthalmic hospitals in India, so that the nuances in treatment parameters and clinical decision-making can be usefully applied to ophthalmic practice. This will ultimately lead to safe and effective treatment delivery in ROP.

Treatment in retinopathy of prematurity (ROP) begins when the disease reaches a stage where vision is threatened. Previous reports have described the screening and establishment of programs for ROP.[1–4] We discuss treatment protocols specifically directed at middle-income countries, where neonatal general anesthesia may not be readily available. However, the same principles also apply to general anesthesia.

*Indications*: The indications for treatment of threshold ROP proposed by the Cryo-ROP study[5] were for those stages that would probably result in adverse visual outcomes in 50% of the eyes.[5,6] [Fig. 1] Concerns were raised regarding delayed treatment using these criteria, and so some physicians also initiated treatment in selected eyes with pre-threshold ROP[1,7] Prethreshold ROP is now divided into high-risk or type 1 and low-risk or type 2, based on eyes that have a risk of 15% or more of adverse outcomes in the early treatment ROP (ETROP) study.[8] Retinal ablation must be considered for any eye with type 1 prethreshold ROP or worse. This includes zone I, any stage of ROP with plus disease; zone I, stage 3 ROP without plus disease; and zone II, stage 2 or 3 ROP with plus disease.

**Figure 1:**
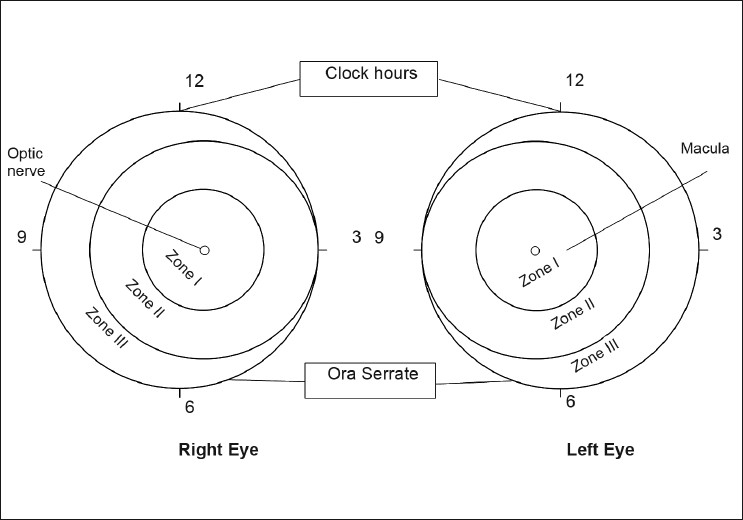
Representation of the classification of retinopathy of prematurity

The anterior limit of zone I is the temporal field of view of a 28 diopter lens placed with one edge on the nasal edge of the optic disc.[9] The remaining part of the retina other than the far periphery will fall into zone II. Any ROP that is continuous and circumferential must be in zones I or II. It must be ensured that there is no ROP in the two nasal-most sectors before the eye is re-categorized as a zone III eye; if this cannot be fully ascertained, the eye is considered to be a zone II eye.[9] Plus disease[9] is defined as at least two quadrants of dilation and tortuosity of the posterior retinal blood vessels [Fig. 2]. Newer methods of classification[9] also include aggressive-posterior ROP (APROP), which need immediate and aggressive treatment.

**Figure 2:**
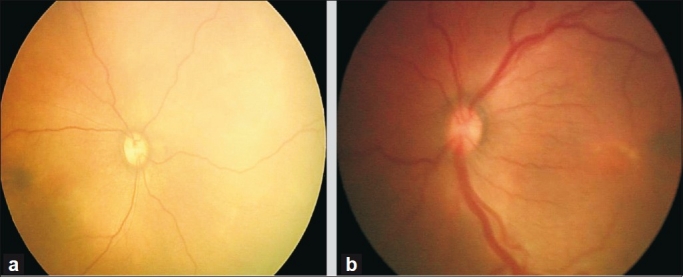
Fundus photo showing normal retinal vessel caliber (a) and plus (b) status

## Practical Tips

Many surgeons are put off by the classification and treatment criteria in clinical practice. Recognizing this, we provide a few simple tips, and also a tabulated version [Table 1] that helps in quick decision-making.

**Table 1:** Current retinopathy of prematurity (ROP) treatment guidelines

Zone I	No Plus	Stage 1	Follow
		Stage 2	Follow
		Stage 3	Treat
Zone I	Plus	Stage 1	Treat
		Stage 2	Treat
		Stage 3	Treat
Zone II	No Plus	Stage 1	Follow
		Stage 2	Follow
		Stage 3	Follow*
Zone II	Plus	Stage 1	Follow*
		Stage 2	Treat
		Stage 3	Treat

Note: Clinical judgment must be applied while using these guidelines. The appearance of plus and new vessels are very important criteria of treatment

* Rare presentations

Treat [Figs. 3a, 3b]: All eyes with plus; eyes without plus having new extraretinal vessels (stage 3), especially if the condition has worsened since the previous visit; APROP eyes urgently and aggressively (involves zone I and posterior zone II).

**Figure 3a:**
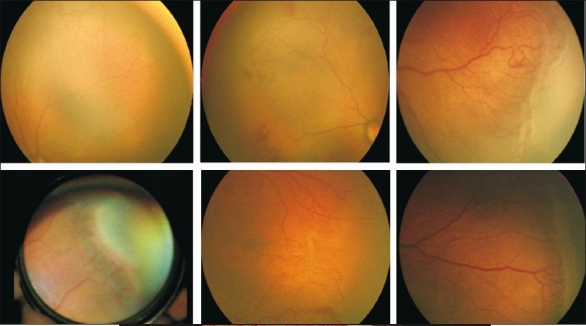
Fundus photos showing various clinical manifestations where laser treatment must be considered

**Figure 3b:**
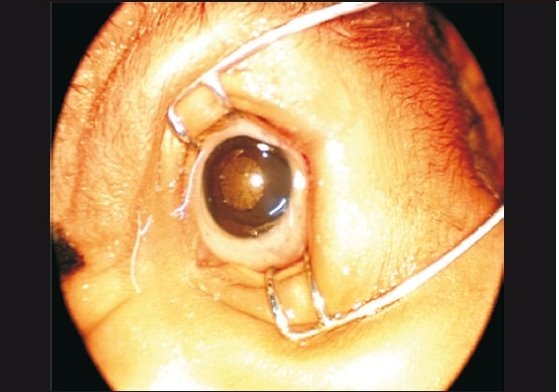
Poorly dilating pupil with engorged iris vessels indicating plus and need for treatment

**Figure 4:**
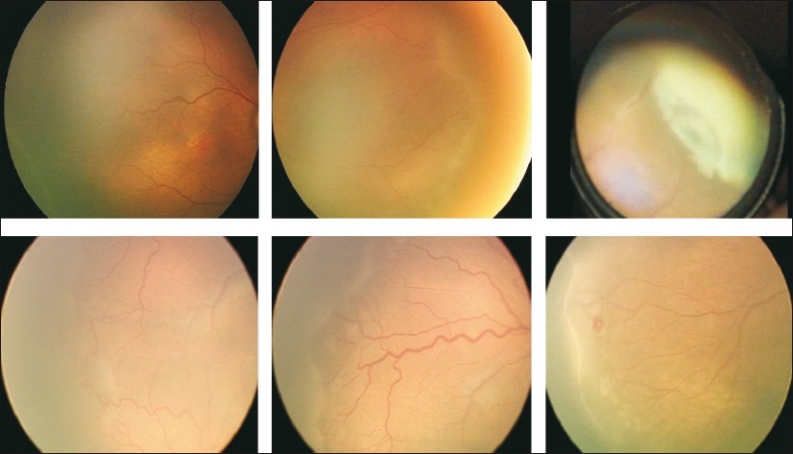
Fundus photos showing various clinical manifestations where laser treatment need not be performed and the child can be followed for spontaneous regression

No treatment [Fig. 4]: for eyes with ROP in zone III; Zone II with no new vessels and no plus. Follow closely, every 7 – 10 days, to watch for regression or progression needing treatment.

Timing and screening criteria for India and middle-income group regions: Start screening between 20 to 30 days of life. One screening session must definitely be completed before day 30 of life (day-30 strategy). Screening must be done for babies born before 34 – 35 weeks of gestational age at birth and/ or birth weight of 2000 g or less.[1,2,3] Earlier (20 days) screening is strongly recommended for babies under 30 weeks, and/or those with weight of less than 1500 g at birth. Delaying the timing of the first screening increases the likelihood of encountering seeing advanced disease, especially in the APROP eyes, and consequently reduces the success rates.

Aim of treatment: To ablate the entire avascular retina as rapidly and as completely as possible with minimum side effects. The effect is usually apparent within one week of adequate therapy.

## Instrumentation, Materials, and Preparation for Laser Therapy

At present, the standard-of-care in ROP is the diode red (810 nanometer wavelength) laser indirect ophthalmoscope. Laser has many advantages over cryotherapy.[10,11] There is less post-treatment pain, adnexal edema, exudative retinal detachment, vitreoretinal traction, and vitreous hemorrhage, due to reduced breakdown of the blood-retinal barrier. Use of topical anesthesia reduces systemic complications such as bradycardia or apnea. Treatment can be done in the incubator itself, even through its walls.[12] In case of inadequate treatment or poor response the laser can be repeated safely at short intervals of one to three days. Visual outcomes reported after laser are better than those after cryotherapy.[13] To avoid needless blindness, cryotherapy may be considered, under general anesthesia, when the laser is not available.[11]

Other materials required include: sterile pediatric eye speculum (Alphonso), wire vectis/ pediatric scleral depressor, sterile cotton-tipped buds, dilating[1] and topical anesthetic eye drops, and sterile Ringer, lactate in a syringe. Ensure that the baby has warm clothing and diapers. Air conditioning must be reduced or special warmers used to avoid hypothermia. Treatment is done either in the Neonatal Intensive Care Unit (NICU) or in an Operating Room equipped with suction apparatus and resuscitation and intubation equipment in the rare event of apnea/ cardiac arrest. A neonatologist or anesthesiologist must be available on call.

Once the decision to treat is taken, the parents and neonatologist are informed. Treatment must be initiated within 24 hours and preferably not later than 72 hours. Treatment must occur in a temperature-controlled, clean environment so that risk of hypothermia, infection, and apnea are minimal. Pupils are dilated using 1% tropicamide and 2.5% phenylepherine instilled twice, 10 minutes apart, at least 30 minutes before the treatment.[1] If tropicamide is not available, 0.5% cyclopentolate may be used instead. The lids must be wiped with cotton to remove spilled droplets. Autoclaved or chemically sterilized (cidex- or Betadine-soaked washed with sterile water) instrument sets must be used. The child must be fed and burped 30 – 60 minutes before treatment if topical anesthesia is used, while four to five hours of fasting is needed if the treatment is under general anesthesia. Pupils may not dilate well in case of severe plus disease [Fig. 3b]. However, do not continue dilating eye drops, especially phenylepherine, as this can cause systemic toxicity and pupils may still not dilate.

## Technique of Laser Under Topical Anesthesia

**Figure 5:**
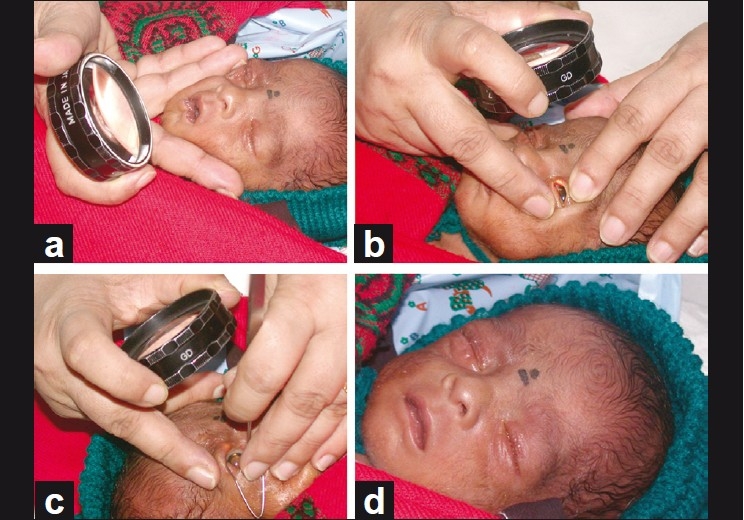
(From a to b) Technique of stabilizing the baby’s chin and evaluating the superior retina; (bottom c to d): stabilizing the forehead to perform laser on the inferior retina. Appearance of the eyes after two hours of laser therapy under topical anesthesia

A restraining band can help hold the baby in place, or an assistant may be called in to help. Topical anesthesia is instilled twice. A sterile pediatric lid speculum is carefully introduced into the conjunctival sac, without touching the cornea. The assistant holds the head while the surgeon stabilizes the chin between the fork formed by the little finger and ring finger, simultaneously holding the 20 diopter lens with the other three digits [Fig. 5].

**Figure 6:**
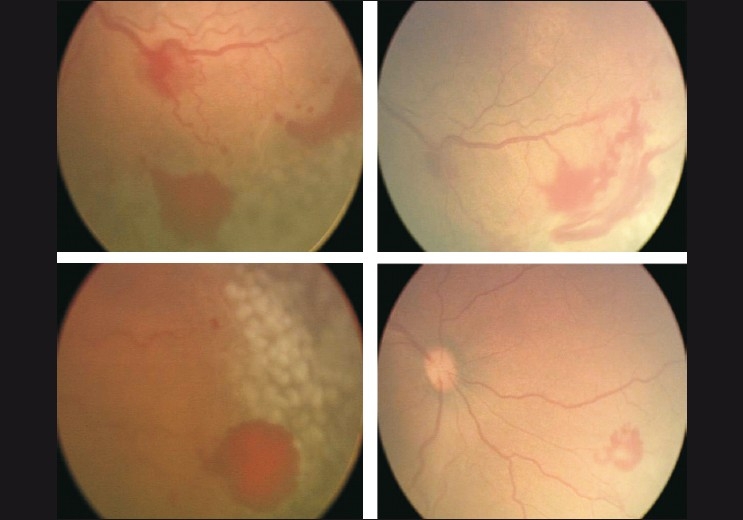
Fundus photos showing (left side) confluent grayish white laser burns going up to the ridge; Inadequate regression (top right) requiring more laser and adequate regression (top bottom) needing only close follow-up seven days after laser

The initial settings on the laser console depend on the fundus pigmentation and area to be treated. Usually, start with 250 milliwatts for 150 milliseconds with the repeat mode set at 300 milliseconds. The treatment must not be faster as this can result in inadequate burns. The intensity must be grayish white rather than white and placement of spots must be nearly confluent[14] [Fig. 6]. The laser power must be varied; less energy must be used for the anterior and superior retina compared to the posterior and inferior retina or to the retina close to the ridge. A wire vectis or pediatric depressor is used to rotate and stabilize the globe, and to indent the anterior retina for laser.

**Figure 7:**
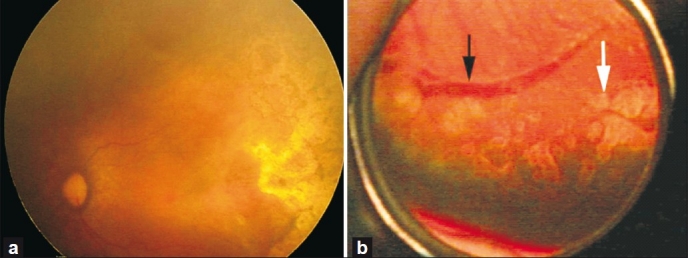
(a) Completely regressed AP-ROP. Note the triangular lasered area at the horizontal raphe, next to the edge of the macula. (b) Nonconfluent laser (white arrow) and non-regressed elevated vessels (black arrow) growing adjacent to the unlasered ‘skip’ area (between the black and white arrows)

While it is essential to treat the entire avascular retina from the ridge/ vascular part of the retina up to the ora for 360 degrees, it is absolutely critical to treat up to the base and all around the ridge and not leave any untreated ‘skip’ areas near the posterior ridge of the avascular retina. ‘Skip’ areas encourage new vessels to grow and prevent active vessels from regressing in that area, resulting in treatment failure [Fig. 7]. In a single session one may place 3000 – 4000 spots in each eye to cover the avascular retina including the enclosed avascular pockets, to adequately treat zone I disease/ APROP eyes. We have not observed any exudative detachment or excessive inflammation in these eyes, provided the intensity of burns is monitored. A smaller number of spots, that is, 1000 – 2000, is needed to manage prethreshold/ threshold zone II with non-APROP eyes.

## Practical Tips

1. Laser in infants’ eyes is more difficult and not the same as in adult eyes and it can last for 60 – 90 minutes. If it takes longer, allow feeding between the sessions for each eye. Due to the steepness and small size of the cornea, peripheral corneal aberrations are higher in infants’ eyes than in adult eyes. Hence, the viewing angle needs to be more vertical. One of the reasons why laser spots cannot be focussed, is the use of a more inclined viewing angle or too much rotation of the globe away from the vertical position of the eye. Reorientation of the viewing angle to a vertical plane and not at an angle, is needed, especially for inferior, nasal, and postequator-inferotemporal areas. Even experienced surgeons have to go through a learning curve in this aspect.
2. For rotation and stabilization of the globe, especially while treating the posterior retina, the instrument (wire vectis / scleral depressor) is held against the forniceal conjunctiva rather than pressing it against the globe. High pressure against the globe causes corneal haziness due to raised intraocular pressure and also leads to undesirable rotation and indentation that make viewing of the posterior retina difficult. In addition, hyphema and vitreous hemorrhage can occur.
3. To visualize the anterior retina, the same instrument is held against the bulbar conjunctiva close to the limbus. In the infants’ eyes the ora serrata is 1.0 mm beyond the limbus. A common mistake committed by beginners is using the depressor 3 – 4 mm beyond the limbus as is usually done in adults; this leads to difficulty in visualizing the ora serrata.
4. While treating the anterior retina, the globe must not be rotated to the extreme ends and an attempt must be made to try to keep it central while using the depressor, to bring the anterior retina forward. The most difficult areas to focus are the inferotemporal mid-periphery in each eye (7 o’clock in right eye and 5 o’ clock in left). Residual new vessels arising from inadequate treatment are not uncommon in these areas.
5. The cornea must be kept adequately irrigated during treatment. Excess water in the fornices is removed by cotton-tipped buds and/ or by tilting the baby’s head.
6. If the pupil is poorly dilated, or it is not possible to focus the laser beam well, an attempt must be made to try and put few spots (5 – 10) in any area even if a little hazy. Simultaneously, firm, mild-to-moderate sustained pressure must be kept on the globe, with the depressor. This often dilates the pupil and allows one to focus the laser rays more accurately.[15]
7. Do not depress too hard and release the pressure on the globe suddenly. This will lead to pupillary constriction and sometimes intraocular hemorrhage. A moderate firm pressure that is slowly released helps to avoid corneal edema and hemorrhage on the one hand and pupillary constriction on the other.
8. Treat the easily accessible areas first, including the area around the base of the ridge and then proceed to the difficult areas. Begin treatment adjacent to the ridge from a particular clock hour, keeping the globe oriented vertically with minimal rotation or indentation if the ridge is posterior to it. More energy (sometimes up to 500 – 600 mW and 200 mSec) may be needed in these posterior areas especially in zone I. Next proceed from the ridge to the mid-periphery with a slight indentation of the globe posteriorly, 4 – 5 mm from the limbus, and gradually move the indenter anteriorly to within 0.5 to 1.0 mm of the limbus, till the laser is complete up to the ora in that clock hour. Proceeding anteriorly, the energy levels need to be reduced by almost 30% to maintain a similar grayish-white intensity of the burns. Then move to the next clock hour and start again. We prefer to start from the temporal quadrants, and then proceed superiorly and superonasally. Later, we return to the temporal quadrant before proceeding inferiorly and inferonasally. Finally we re-treat the triangular macular-edge area along the horizontal raphe temporally, as this area is difficult to treat. Check all around for any untreated skip areas at the end. In AP-ROP be sure to treat along the posterior wavy ridge, which is sometimes missed, especially when the child presents late and some vessels have already gone beyond the ridge.
9. Ensure that while treating anterior retina, burns are placed up to but not beyond the ora. The ora serrata is identified by a white area with a leathery/ shiny texture bordered by the dull brown area of the ciliary body. Burns over the ciliary body will result in cyclophotocoagulation and cause serious complications such as, hypotony, cataract, and uveitis.
10. Avoid heavy confluent spots anteriorly at the point of entry of the long posterior ciliary vessels and nerves; this is postulated to cause hypotony and cataract. Mild intensity burns can be placed in these areas.

Throughout the procedure, an infant pulse oxymeter must monitor the baby. Ensure that the baby is making clear vocal sounds with no secretions in the throat. A very quiet child must arouse immediate suspicion of apnea/ cardiac arrest. This is a rare, but serious event, and needs to be constantly watched out for. A baby who is crying slowly and moving the limbs is reassuring. At the end of the procedure, wipe the eyelids with a wet swab to reduce edema. Instill a drop of antibiotic; ensure that the child is breathing well. Hand the child over to the parents/ nursing staff.

Postoperative instructions: The eyelids and conjunctiva generally have moderate edema, erythema and subconjunctival hemorrhage for three to four days. NICU staff and parents must be told of these adnexal effects to avoid alarm. Topical steroids are prescribed thrice a day to reduce any inflammation and to decrease the risk of post-laser posterior synechiae. In case the child is no longer in the NICU, it is important to ensure that the child is adequately nourished before being discharged. Usually, the baby is a little exhausted and may not feed for 15 – 30 minutes after the laser treatment.[4] After a short rest, encourage the child to initiate feeding by stimulating the soles of the feet. However, in case of any doubt or difficulty in feeding the child, do not force-feed as this could cause the baby to aspirate. If this happens, immediately transfer the baby to a warm environment with oxygen support if needed. Inform the pediatrician/ NICU as they may need to manage hypoglycemia, hypoxia or apnea. The anesthetist or NICU staff must monitor the baby until he/ she is fed and is stable. Adhering to these practical tips will go a long way in preventing life-threatening problems and ensure a smooth postoperative period.

## Frequently Asked Questions

**Figure 8:**
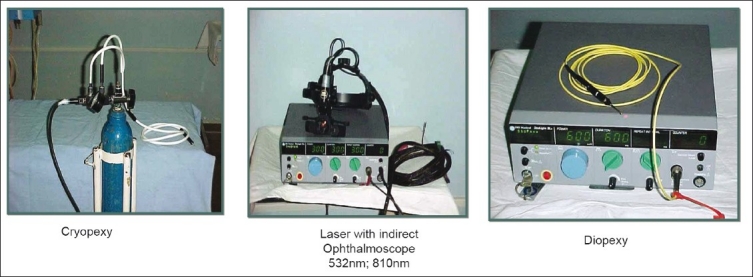
Instrumentation for ROP retinal ablation

1. Why is general anesthesia not used in all cases? Ideally it would be wonderful to have general anesthesia for all ROP treatments. However difficulties with such a protocol include non-availability of expert neonatal anesthesiologists, difficulty in administering anesthesia frequently, and anesthesia-related morbidity and mortality in view of frequent problems such as anemia, sepsis, pneumonia, chronic lung insufficiency, hyperbilirubinemia, and so on. Moreover, our experience indicates that topical anesthesia suffices and allows the laser to be administered without any difficulty even in AP-ROP eyes.
2. Can we use frequency double 532 green laser in ROP? In eyes with adequately dilating pupils and no abnormal vessels in the anterior segment, green laser wavelengths can be used effectively. With adequate laser expertise, even eyes with tunicosa vasculosa lentis can be treated safely. The theoretical disadvantage of green laser is that it can get absorbed by the abnormal blood vessels around the crystalline lens and cause lenticular opacities, especially in the hands of beginners.
3. What must be done if the view gets hazy during laser therapy? The view gets hazy due to corneal edema, secondary to excessive pressure on the globe, from using distilled water for irrigation, or due to hyphema from dilated iris vessels and sometimes due to intraoperative vitreous hemorrhage. Often, the corneal edema will clear a few minutes after removal of the eyelid speculum. If this does not happen, it is better to perform laser on the next day or at the next sitting. If hyphema occurs, wait for four to eight hours until it clears, after which the laser can be completed. Vitreous hemorrhage is an ominous sign and indicates advanced disease. One must ablate as far as possible with the indirect ophthalmoscope laser along with supplemental cryoablation or diopexy ablation [Fig. 8].

Follow-up after laser: In patients with less severe disease, often in anterior zone II, where adequate laser treatment has been done in the first session, one must re-evaluate after seven days and check for signs of regression. If adequate regression has not occurred or focal areas of active new vessels are seen, treatment is added to the areas skipped and around the active area. In zone I or APROP cases or in eyes with media haze, one session of treatment is usually inadequate. In such eyes, re-evaluation and completion of laser ablation must be done every three to four days until complete regression is seen.

### Signs of regression

All signs of plus disease must completely regress before discontinuing treatment sessions. Signs that indicate disease that have reached a quiescent phase of acute ROP [Fig. 7] after retinal ablation are shown in Table 2.

**Table 2:** Signs of Regression of retinopathy of prematurity

Media clear
Pupil dilates fully and readily
No new vessels in the iris
No new vessels in the retina
All retinal / preretinal and vitreous hemorrhages cleared
Regression of dilatation and tortuosity of retinal vessels
No increase in retinal traction manifested by disc / macula / arcade drag
No elevation of retina / ridge at or posterior to area of laser
Feeder vessels to area of active new vessels / hemorrhages / elevated ridge and so on, achieve normal caliber
Demarcation between laser-treated and normal retina is quiet and flat in terms of vasculature, with adequate scar effect of the laser

Plus disease can be masked if the child is on oxygen, is anemic, if too much pressure is put on the globe or if a 20-diopter lens is not used (for instance if a 28 or 30 diopter lens is used) to assess plus. Lens sparing vitrectomy must be considered early enough in case traction/ ridge elevation of more than four clock hours has developed. The surgery has a better outcome in cases where laser has failed, if it is done before the baby reaches 41 weeks of post-conceptional age. A close follow-up is needed for eyes with traction, as retinal detachment is not uncommon and early surgery protects against blindness.

*Long Term Follow-up*: Once acute phase ROP completely regresses, the child is followed up (along with the pediatric ophthalmologist) for visual development, strabismus, refraction, anisometropia-amblyopia, and retinal and lenticular status at regular intervals. The details of this follow-up are beyond the scope of the present communication and are published elsewhere.

### Expected outcomes of Retinal ablative treatment:

Treatment at the threshold stage[16] had a 20% reduction in absolute risk for an unfavorable outcome, defined as a fold through the macula, and partial or total retinal detachment. However, less than 20% of the eyes with favorable outcome achieved a visual acuity of 20/40 or better.[16,17] Grading acuity at nine months post treatment at the prethreshold stage showed a reduction in unfavorable outcomes with earlier treatment, from 19.8 to 14.3%.[13] The goal of achieving 20/40 or better in 80% or more eyes is possible[17] with appropriate and timely treatment, as outlined in the current article and depicted practically in a video.[18]

## Conclusion

The critical factors to ensure better outcomes in ROP are to screen early, follow-up closely, watch out for plus and/ or new vessels and treat such eyes vigorously for full avascular retina ablation. ROP is a continuous race against time with a very small window of opportunity of 7 – 10 days, where optimum treatment outcomes with least complications can be achieved. Safe and effective treatment techniques must be applied to achieve outcomes that are both optimal and satisfying in the long term.
